# Supplementary material for: Amyloid β-Oligomers Inhibit the Nuclear Ca2+ Signals and the Neuroprotective Gene Expression Induced by Gabazine in Hippocampal Neurons
Source: Antioxidants (Basel). 2023 Nov 6;12(11):1972. doi: 10.3390/antiox12111972 (PMC10669355; doi:10.3390/antiox12111972)
Supplement: Supplementary file 1 [file antioxidants-12-01972-s001.zip › Supplementary Material.pdf]

Supplementary Material:

.

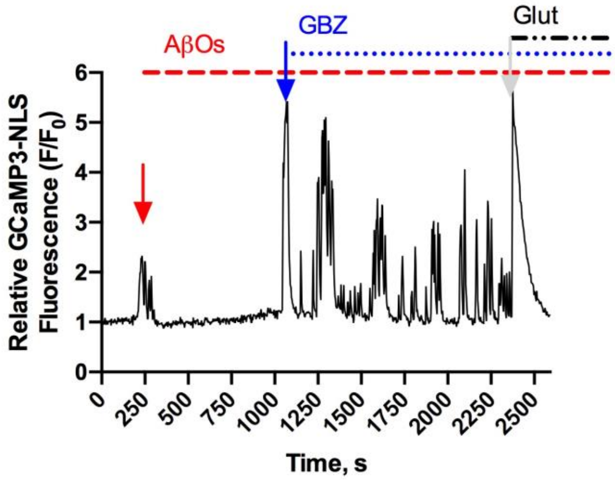

Supplementary Figure S1. Treatment with AβOs for 15 minutes does not disrupt the transient nuclear Ca^{2+} signals induced by GBZ in pyramidal hippocampal neurons. Neurons were treated with AβOs (500 nM) at the microscope stage at the time indicated by the red arrow, and the relative change in fluorescence values with time (F_{max}-F_0/F_0) was recorded for 15 minutes in the nucleus of neurons expressing GCaMP3-NLS before GBZ addition (red dashed line). After addition of 5 μM GBZ (blue arrow), the typical oscillatory and synchronous Ca^{2+} signals induced by GBZ can be observed (blue dashed line). Also, the record shows that addition of 10 μM glutamate causes a high intensity transient of Ca^{2+} in the nucleus of these neurons. A representative graph of three experiments performed in independent cultures is shown

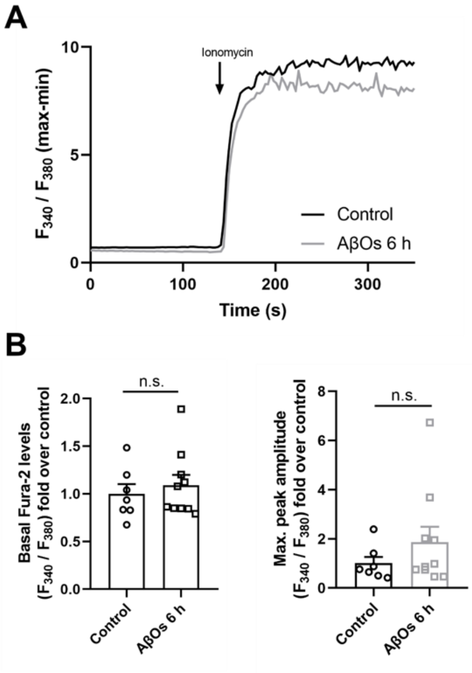

Supplementary Figure S2. Cytoplasmic Ca^{2+} levels in primary hippocampal neurons. A) Representative records of the relative change in Fura-2 fluorescence (F_{340}/F_{380}) with time, displayed by control neurons or neurons treated for 6 h with AβOs (500 nM). The arrow indicates the time of ionomycin addition. **B**) The basal Fura-2 fluorescence levels and the maximal peak amplitude following ionomycin addition were quantified. The average values obtained from a minimum of seven experiments performed in independent cultures show no significant differences between control neurons and neurons preincubated with AβOs. Data are expressed as Mean ± SE. Statistical analysis was performed with Mann-Whitney Test. N.s. not significant.

Supplementary Table S1: Sequence of primers used to determine the mRNA levels of the RyR2, BDNF exon IV, Npas4, NqoI and β-actin. (ref ncbi)

**Gene**	**NCBI Reference Sequence**	**forward (5' → 3'):**	**reverse (3' →5'):**
RyR2	NM_001191043.3	AATCAAAGTGGCGGAATTTCTTG	TCTCCCTCAGCCTTCTCCGGTTC
Npas4,	NM_153626.1	CCGCCATGCAATTTCCACTA	CGGTCCCCAAGGTTCTAGACT
Nqo1	NM_017000.3	CTTTCCAGAATAAGAAGACCTTGC	TGCTGTACACCAGTTGAGGTT
β-actin	NM_031144.3	TCTACAATGAGCTGCGTGTG	TACATGGCTGGGGTGTTGAA

Supplementary Video S1: Treatment with AβOs disrupts the transient nuclear Ca^{2+} signals induced by GBZ in hippocampal neurons.

https://www.dropbox.com/home/Andrea%20Paula%20Lima/Papers/Paper%20Pedro%20AbOs%20Nuclear%20Calcium?preview=VIDEO-2023-08-28-20-27-22.mp4
